# Antibiotic-loaded calcium sulfate in clinical treatment of chronic osteomyelitis: a systematic review and meta-analysis

**DOI:** 10.1186/s13018-022-02980-2

**Published:** 2022-02-19

**Authors:** Xiangwen Shi, Yipeng Wu, Haonan Ni, Mingjun Li, Chaoqun Zhang, Baochuang Qi, Mingjie Wei, Teng Wang, Yongqing Xu

**Affiliations:** 1 Kunming Medical University, No. 1168 Yu Hua Street, Kunming, 650000 China; 2 Institute of Traumatology and Orthopedics, 920th Hospital of Joint Logistics Support Force, PLA, Kunming, 650000 China

**Keywords:** Chronic osteomyelitis, Calcium sulfate, Tobramycin, Gentamicin, Vancomycin, Meta-analysis

## Abstract

**Background:**

Present work was aimed to gather accessible evidence on the eradication rates and related postoperative complications of antibiotic-loaded calcium sulfate (CS) as an implant in the treatment of chronic osteomyelitis (COM).

**Methods:**

Databases including PubMed, EMBASE, Medline, Ovid and Cochrane library were searched from their dates of initiation until November 2021. Two independent authors scrutinized the relevant studies based on the effectiveness of radical debridement combined with antibiotic-loaded CS for COM; data extraction and quality assessment of the Methodological Index for Non-Randomized Studies (MINORS) criteria were also performed by the authors. In addition, clinical efficacy mainly depended on the evaluation of eradication rates and complications, and all the extracted data are pooled and analyzed by STATA 16.0.

**Results:**

A total of 16 studies with 917 patients (920 locations) were recruited, with an overall eradication rate of 92%. Moreover, the overall reoperation rate, overall refracture rate, overall delayed wound healing rate, and the rate of aseptic wound leakage were 9.0%, 2.0%, 20.0%, and 12.0%, respectively. Moreover, the choice of tobramycin-loaded CS or vancomycin combined with gentamicin-loaded CS did not affect the eradication rate, and the incidence of postoperative complications in COM patients (all $P>0.05$). The general quality of the included studies was fair.

**Conclusions:**

Our meta-analysis indicated that the overall eradication rate of COM treated with antibiotic-loaded CS was 92%. Delayed healing is the most common postoperative complication. The choice of tobramycin-loaded CS or vancomycin combined with gentamicin-loaded CS did not affect the eradication rate and the incidence of postoperative complications in COM patients.

## Introduction

Chronic osteomyelitis (COM) is defined as a significant and long-standing infection of bone tissue, which lasting more than several months or even years and involved in any bone including periosteum, bone marrow and surrounding tissue. Most infections occur after trauma, surgery or secondary to vascular and neurologic insufficiency (e.g. diabetic foot ulcers), characterized by persistent bacteria, low-grade inflammation, and the prevalence of fistula and dead bone [1, 2]. With the improvement of diagnosis and population aging, COM incidence has apparently increased [3, 4]. In 2004, nearly 600,000 artificial joint replacements and 2 million internal fixation of fracture caused more than 110,000 infections finally in the USA [5]. According to related research estimation, the treatment of implant-related COM will cost $1.62 billion by 2020 in US hospitals, and impose a substantial financial burden on patients and society [6]. Furthermore, infected chronic diabetic foot ulcers may lead to diabetic foot osteomyelitis, which will increase the mortality and risk of amputation [7, 8]. The recurrent and persistent infection is a challenging condition for both the physician and the patient.

The mainstays of COM treatment are radical debridement, then supplemented with targeted antimicrobial therapy (local and/or systemic) for long time. A recent study suggested that the combination of antibiotics and surgery seems to be more effective than any single method [9]; despite these measures, infection recurred in 20% patients [10]. Due to the vascular injury in infected bone, it is difficult to achieve effective local antibiotic concentration by oral or intravenous antibiotic treatment under local ischemia; and the limited biofilm penetration makes the treatment of COM more difficult [1, 11, 12]. Therefore, the focus of antibiotic therapy is to use the local drug delivery system to avoid the potential toxicity caused by systemic administration, while providing a sustained high concentration at the infection site [13]. It is very important for the long-term treatment of osteomyelitis, especially for the relief of symptoms [14].

Calcium sulfate (CS) is a kind of biodegradable material with low immunoreactivity, easy reabsorption and good tolerance, which has been proved to be effective and safe as an antibiotic carrier in the past two decades [15–18]. CS is as effective as polymethyl methacrylate (PMMA) as an antibiotic carrier in bone infections [19]. However, Chang et al. [20] compared the efficacy of debridement plus CS combined with tobramycin and simple debridement, the results showed that the curative effect was not obvious, and the success rate of bone osteomyelitis was 80% and 60%, respectively. In terms of complications, several experiments indicated that CS products have transient cytotoxicity, leading to inflammation, within the first 60 days after implantation, CS causes inflammation in the surrounding tissue. After 60 days, the inflammation in the affected bone subsided, but the inflammation in the surrounding soft tissue did not subside, and the problem of surgical wound healing followed [21]. Due to the inconsistency on the properties of CS materials and surgical procedures, aseptic wound leakage and re-fracture have gradually got increasing attention, which may become the causes of reoperation or infection recurrence [22]. Jiang et al. [23] found that the incidence of aseptic wound leakage after antibiotic-loaded CS implantation treated COM was very high. Therefore, in this review, we comprehensively evaluate the efficacy and complications of CS loaded with multiple antibiotics in the treatment of COM (including delayed healing, reoperation, refracture and aseptic wound leakage). Another controversial issue is the efficacy of different antibiotic-loaded CS. The most common antibiotics used with CS are tobramycin and vancomycin, the former effectively reduces the chance of prosthetic infection and prevents biofilm formation and colonization of bacteria such as Methicillin-resistant Staphylococcus aureus [24]; the latter combined with gentamicin can cover a broad spectrum of both gram-positive and gram-negative bacteria [25]. At present, a variety of products have been sold in the market [26], and a number of clinical studies have been conducted; but it is still unclear whether there are differences in the efficacy and complications of different antibiotics in the treatment of COM.

The major purpose of this meta-analysis was to investigate the eradication rates and local complications of antibiotic-loaded CS in treating COM patients. Second, we explored difference of curative effect and related postoperative complications between two antibiotic regimens. To provide reference for orthopedic surgeons using antibiotic-loaded CS in the treatment of COM.

## Material and methods

**Fig. 1:**
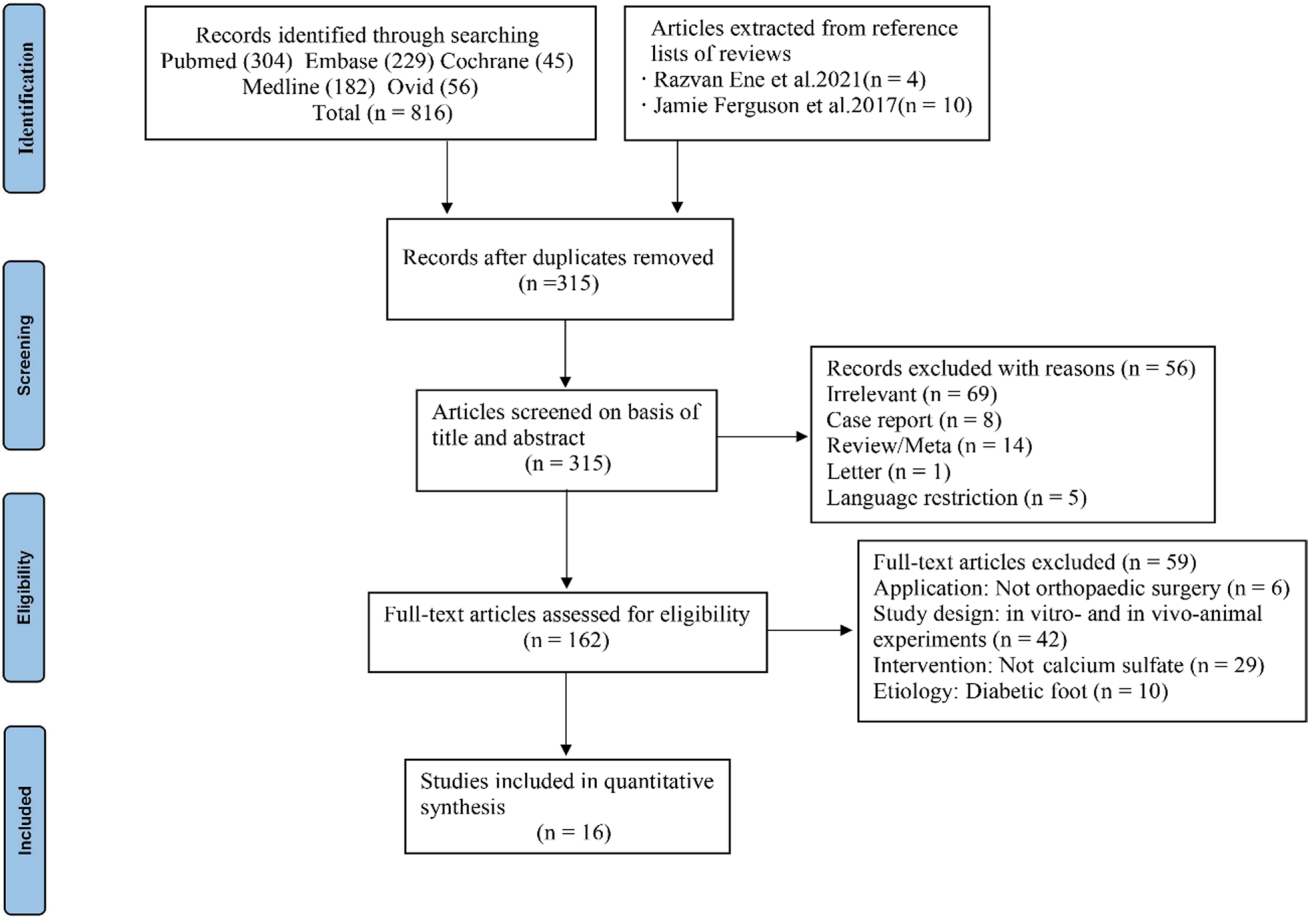
PRISMA flow diagram of the study selection process

This study was presented in accordance with the PRISMA (Preferred Reporting Items for Meta-Analyses and Systematic Reviews) [27]. The PRISMA checklist is in the additional file, and the flow diagram of literature screening process is shown in Fig. 1. Ethical approval was not required.

### Search strategy

Online databases were used to identify eligible studies, we searched all articles in PubMed, EMBASE, Medline, Ovid and Cochrane Library up to November 2021. We also performed a supplementary search in the Ovid database to avoid missing articles. In addition, a manual search of references and similar documents of identified articles was also performed to determine potential relevance. Medical subject heading (MeSH) and Embase Tree tool (EMTREE) were used to guide the choice of appropriate search terms in all databases. We performed a search using the strategy (“osteomyelitis” OR “osteitis” OR “bone infection” OR “osteoarticular infection”) AND (“calcium sulfate” OR “calcium sulphate”). The last search was performed on November 15, 2021.

### Eligibility criteria

The study selection criteria were established in accordance with the PICOS strategy (Patients, Intervention, Comparison, Outcomes, Study design): population—Patients with COM who underwent radical debridement and antibiotic-loaded CS; intervention—Operation with antibiotic-loaded calcium sulfate(including tobramycin, vancomycin and gentamicin); comparison—none; outcomes—Eradication rate and complications of failure, including reoperation, refracture, and delayed healing; study design—consecutive case–control studies or case series. Several eligibility criteria were applied in the present study. Only case series and consecutive case–control studies regarding the clinical application of antibiotic-loaded CS in the treatment of COM were eligible for inclusion, case reports, review articles, meta-analysis, animal researches, or unpublished studies were excluded. Articles written in a language other than English or German were excluded. Publication, studies involving patients with COM due to any causative mechanism except diabetic ulcer were eligible for inclusion. In order to be included, each eligible report had to contain at least one of the outcomes of interest. The main outcome of interest was eradication rates of infection, but studies that described postoperative local complications (wound-healing problems, aseptic wound leakage, etc.) were also eligible for inclusion. Patient selection was not restricted by age, gender, or other personal characteristics.

### Data collection

All the identified studies were selected according to the title and abstract by two independent authors (XW.S. and YP.W.). Extracted data were performed by 2 other authors independently (NH.N. and MJ.L.) and they screened articles with potential relevance through overall assessment based on the inclusion and exclusion criteria. The extracted data was summarized into a table, divided into three parts: (1) specific information of eligibility studies, including the authors’ names, year of publication, region of research, and study design; (2) basic information of each case, including age, total number of cases and gender, follow-ups, treatment, bacterial culture and application of antibiotics; (3) postoperative condition of each patient including failure, refracture, reoperation, delayed wound healing, local complications and other adverse events. For studies with incomplete or unclear data, we attempted to contact the authors for details. If any disagreement existed, a third reviewer participated and reached consensus.

### Quality assessment

The Methodological Index for Non-Randomized Studies (MINORS) criteria was used to evaluate the quality of the included studies independently by two authors, which was established to assess the quality of comparative and noncomparative studies [28]. The highest score is 24 for comparative studies and 16 for noncomparative studies. Specific rating criteria are as follows: in noncomparative studies, scores of 0–4 showed to very low quality, 5–7 showed to low quality, 8–12 showed to fair quality, and ≥ 13 showed to high quality; in comparative studies, scores of 0–6 showed to very low quality, 7–10 showed to low quality, 11–15 showed to fair quality, and ≥ 16 showed to high quality.

### Statistical analysis

Stata 16.0 was used to pool the all results of the included studies were for the meta-analysis. A chi-squared-based Q statistical test was used to estimate the statistical heterogeneity. The degree of heterogeneity for each included study was quantified using the *I*^2^ statistic. When *P* > 0.1 and/or *I*^2^ < 50%, the heterogeneity was evaluated to be low, and a fixed-effects model was used for the meta-analysis. Otherwise, a random-effects model was used. We calculated the postoperative eradication rates and incidences of complications in COM patients, as well as its 95% confidence interval (CI) for each study. Further, the pooled rates were calculated and publication bias was evaluated with a funnel plot. All results are presented in the form of forest plots and tables, and *P* < 0.05 was considered statistically significant.

## Results

### Search results

Initially, 816 articles were identified by searching, and two authors selected 162 studies by reading the title and abstract. Finally, through scrutinizing the full text and performing a manual search, a total of 16 articles conforming to requirements were included in our study. The implants used in one study [25] were self-configured. After careful research and discussion, we decided to include this study because it had a large sample size and a long follow-up period, which provided specific information on the configuration of antibiotics-loaded CS. In addition, there were two studies [29, 30] from the same medical center at different times, but after careful comparison with the inclusive information of patients, we found that duplicate patients were not included, so we considered including these studies. We also contacted the author for more detailed patient information for comparison.

### Study characteristics

All the included 16 studies were published between 2002 and 2021 [15, 23, 25, 29–41]. Of the included studies, three consecutive case–control studies were retrospective in nature; the remaining were case series, including 2 prospective studies and 11 retrospective studies. Of the 16 publications, 7 were from China, 2 were from USA, 2 was from UK, and Germany, France, Spain, Italy, and Egypt each account for one case. The implants used in these studies included vancomycin, gentamicin-impregnated CS and tobramycin-impregnated CS. After the antibiotics-loaded CS was implanted, different surgical methods were performed according to the situation, including external fixation, skin or muscle flap transplantation, and skin grafting. Moreover, all the COM patients met the diagnostic criteria: obvious local symptoms, changes in imaging examination, and elevated inflammatory markers. Table 1 lists the COM patients’ characteristics in this meta-analysi s.

**Table 1:** Characteristics of included studies

Study cohort	Study region	Study Design	Patients(I/C)	Sex(M/F)	Age (years)	Follow-up (months)	Location	Culture Results	Intervention	Local complications	Other Adverse Events	Outcomes	MINORS
Humm, 2014 [31]	UK	Retrospective outcome study	21/NA	18/3	49 (28–88)	16 (6–25)	21 tibia	4 Staphylococcus aureus, 4 Coagulase-negative Staphylococci, 4 Polymicrobial, 3 Negative, 6 Other organisms	Debridement, tobramycin-impregnated calcium sulfate	7 aseptic wound leakage, 5 pin-tract infection	a transient acute kidney injury	Eradication rate, reoperation rate, delayed healing rate, rate of aseptic wound leakage	8
Andreacchio, 2019 [32]	France	Retrospective outcome study	12/NA	8/4	10.3 (2–15)	24–72	3 tibia,4 femur, 2 humerus,1 clavicle,1radius,1 IV Metatarsal	3 Methicillin-resistant Staphylococcus aureus, 7 Negative (other NS)	Debridement, tobramycin-impregnated calcium sulfate	None	None	Eradication rate, reoperation rate, refracture rate, rate of aseptic wound leakage	9
Ferguson, 2014 [33]	UK	Retrospective outcome study	193(195 locations)/NA	150/43	46.1 (16.1–82)	44.44 (15.6–85.2)	88 tibia, 73 femur, 10 humerus, 6 ankle, 5 radius, 4 knee fusion, 4 pelvis, 3 calcaneum, 1 ulna, 1 forefoot	49 Methicillin-sensitive Staphylococcus aureus, 10 Coagulase-negative staphylococci, 7 Methicillin resistant Staphylococcus aureus, 4 Escherichia coli, 4 Enterobacter cloacae (other NS)	Debridement, tobramycin-impregnated calcium sulfate	30 aseptic wound leakage, 9 collection of fluid and 9 refracture	7 death (other reason)	Eradication rate, reoperation rate, refracture rate, delayed healing rate, rate of aseptic wound leakage	13
McKee, 2002 [15]	Canada	Prospective outcome study	25/NA	15/10	43 (27–69)	28 (20–38)	8 tibia, 6 femur, 3 ulna, 1 humerus	9 Staphylococcus aureus, 4 Staphylococcus epidermidis, 4 Pseudomonas aeruginosa, 2 Enterobacter cloacae, other polymicrobial infections	Debridement, tobramycin-impregnated calcium sulfate	8 aseptic wound leakage, 3 refracture, 2 persistent nonunion, 1 superficial wound necrosis, 1 hypertrophic nonunion	NR	Eradication rate, reoperation rate, refracture rate, delayed healing rate, rate of aseptic wound leakage	9
Gitelis, 2002 [34]	USA	Retrospective outcome study	6/NA	3/3	50 (26–85)	28 (18–40)	3 tibia, 3 femur	5 Staphylococcus aureus, 1 Polymicrobial infections	Debridement, tobramycin-impregnated calcium sulfate	None	NR	Eradication rate, reoperation rate, refracture rate, delayed healing rate, rate of aseptic wound leakage	8
Qin, 2020 [30]	China	Retrospective outcome study	33/NA	26/7	44.5 (17–67)	35.9 (12–75)	33 calcaneum	8 Staphylococcus aureus,6 Pseudomonas aeruginosa, 2 Enterococcus faecalis, 2 Proteus mirabilis,2 Enterobacter cloacae,10 negative (other NS)	Debridement, vancomycin and gentamicin-impregnated calcium sulfate	13 aseptic wound leakage	1 death (cardiovascular disease)	Eradication rate, reoperation rate, refracture rate, delayed healing rate, rate of aseptic wound leakage	7
Ferrando, 2017 [35]	Spain	Retrospective comparative study	13/12	9/4	48 (17–67)	22 (16–29)	6 tibia, 4 calcaneum, 2 femur, 1 humerus	5 methicillin-sensitive Staphylococcus aureus, 2 Methicillin-resistant S.aureus, 1 Pseudomonas aeruginosa, 1 Enterobacter cloacae, 1 Streptococcus agalactis,1 Escherichia coli,1 Polymicrobial infections	Debridement, vancomycin and gentamicin-impregnated calcium sulfate	1 hematoma,1 mild seroma	None	Eradication rate, reoperation rate, delayed healing rate, rate of aseptic wound leakage	15
Badie, 2019 [36]	Egypt	Prospective outcome study	30/NA	25/5	26.2 (17–53)	> 12	14 tibia, 11 femur, 2 radius, 2 humerus, 1 ulna	15 S. aureus, 3 methicillin resistant S. aureus, 2 Klebsiella Pneumoniae, 2 Escherichia coli, 2 Proteus mirabilis, 1 Salmonella, 1 Streptococcus, 2 polymicrobial infections, 2 negative	Debridement, vancomycin and gentamicin-impregnated calcium sulfate	1 refracture	NR	Eradication rate, reoperation rate, refracture rate, delayed healing rate	11
Jiang, 2020 [23]	China	Retrospective outcome study	34/NA	27/7	41 (3–67)	26 (12–68)	34 calcaneum	5 Pseudomonas aeruginosa, 2 Enterobacter cloacae, 2 Staphylococcus aureus (other NS)	Debridement, vancomycin and gentamicin-impregnated calcium sulfate	11 aseptic wound leakage	1 death (other reason)	Eradication rate, reoperation rate, refracture rate, delayed healing rate, rate of aseptic wound leakage	9
Zhou, 2020 [37]	China	Retrospective outcome study	42(43 locations)/NA	24/18	43.7 (23–74)	42.8 (12.8–77.5)	24 left tibia, 19 right tibia	11 Staphylococcus aureus, 3 Pseudomonas aeruginosa, 1 Polymicrobial infections	Debridement, vancomycin and gentamicin-impregnated calcium sulfate	13 paseptic wound leakage, 4 slight pain after a long-distance walk, 4 limb weakness or discomfort, 1 slight claudication	NR	Eradication rate, reoperation rate, refracture rate, delayed healing rate, rate of aseptic wound leakage	8
Qin, 2018 [29]	China	Retrospective comparative study	35/NA	26/9	38 (18–60)	33.7 (25 ~ 41)	35 tibia	15 Staphylococcus aureus, 5 Escherichia Coli, 3 Pseudomonas Aeruginosa, 2 Serratia Marcescens, 2 Acinetobacter Baumannii, 2 Klebsiella Pneumoniae, other negative	Debridement, vancomycin and gentamicin-impregnated calcium sulfate	8 pin-tract infection, 3 knee stiffness	None	Eradication rate, reoperation rate, delayed healing rate, rate of aseptic wound leakage	7
Gramlich, 2017 [38]	Gemany	Retrospective outcome study	93/NA	59/34	62 (11–84)	11 (6–22)	35 femur, 28 tibia, 7 fibula, 5 humerus, 5 hip joint, 4 Radius, 3 talus,, 3 pelvis (other NS)	27 Staphylococcus aureus, 19 Staphylococcus epidermidis, 8 Pseudomonas aeruginosa, 5 Escherichia coli, 3 Klebsiella Pneumoniae (other NS)	Debridement, vancomycin and gentamicin-impregnated calcium sulfate	NR	NR	Eradication rate	6
Ruan, 2021 [39]	China	Retrospective outcome study	35/NA	25/10	54 (34–82)	24–60	35 tibia	4 Staphylococcus aureus, 2 Klebsiella Pneumoniae, 2 Streptococcus	Debridement, vancomycin and gentamicin-impregnated calcium sulfate	5 anterolateral numbness of the iliac thigh, 2 relapse, 2 hematocele in the iliac bone area, 1 nonunion, 1 aseptic exudate	NR	Eradication rate, reoperation rate, refracture rate, delayed healing rate, rate of aseptic wound leakage	10
Gauland, 2011 [25]	USA	Retrospective outcome study	323/NA	NR	NR	60	Lower-Extremity (NS)	NR	Debridement, vancomycin and gentamicin-impregnated calcium sulfate	NR	NR	Eradication rate, reoperation rate, delayed healing rate, rate of aseptic wound leakage	8
Sun, 2017 [40]	China	Retrospective outcome study	12/NA	7/5	54 (16–72)	10.8 (6–18)	12 jaw	3 Staphylococcus aureus, 2 β-hemolytic streptococcu, 1 Escherichia coli, 1 Streptococcus viridans (other NS)	Debridement, vancomycin-impregnated calcium sulfate	2 aseptic wound leakage	None	Eradication rate, reoperation rate, delayed healing rate, rate of aseptic wound leakage	7
Zhao, 2020 [41]	China	Retrospective comparative study	10/21	10/0	48 (28.98–67.42)	21.7 (15.8–27.6)	5 femur, 5 tibia	4 Staphylococcus aureus, 4 Negative (other NS)	Debridement, vancomycin-impregnated calcium sulfate	3 aseptic wound leakage	None	Eradication rate, reoperation rate, delayed healing rate, rate of aseptic wound leakage	14

NR, not reported; NS, not specified

### Quality assessment

The data were extracted and evaluated independently by two authors from the included studies. For 8 noncomparative studies, the results showed that average MINORS score was 8.5 (range from 6 to 13), suggesting fair quality. The remaining two comparative studies had scores of 14 and 15, both suggesting fair quality. Overall, the methodological quality of the included studies was moderate (Table 2).

**Table 2:** Methodological quality of included studies

Items methodological items for non-randomized studies	Humm, 2014 [31]	Andreacchio, 2019 [32]	Ferguson, 2014 [33]	McKee, 2002 [15]	Gitelis, 2002 [34]	Qin, 2020 [30]	Ferrando, 2017 [35]	Badie, 2019 [36]	Jiang, 2020 [23]	Zhou, 2020 [37]	Qin, 2018 [29]	Gramlich, 2017 [38]	Ruan, 2021 [39]	Gauland, 2011 [25]	Sun, 2017 [40]	Zhao, 2020 [41]
1. A clearly stated aim: the question addressed should be precise and relevant in the light of available literature	2	2	2	2	2	1	2	2	1	2	1	2	2	2	2	2
2. Inclusion of consecutive patients: all patients potentially fit for inclusion (satisfying the criteria for inclusion) have been included in the study during the study period (no exclusion or details about the reasons for exclusion)	0	2	2	0	1	0	1	0	0	0	0	0	0	0	0	0
3. Prospective collection of data: data were collected according to a protocol established before the beginning of the study	0	0	0	2	0	0	0	2	0	0	0	0	0	0	0	0
4. Endpoints appropriate to the aim of the study: unambiguous explanation of the criteria used to evaluate the main outcome which should be in accordance with the question addressed by the study. Also, the endpoints should be assessed on an intention-to-treat basis	2	1	2	2	1	2	2	2	2	2	2	1	2	2	2	2
5. Unbiased assessment of the study endpoint: blind evaluation of objective endpoints and double-blind evaluation of subjective endpoints. Otherwise the reasons for not blinding should be stated	1	1	2	0	0	0	1	1	2	0	0	0	2	0	0	0
6. Follow-up period appropriate to the aim of the study: the follow-up should be sufficiently long to allow the assessment of the main endpoint and possible adverse events	1	1	2	1	2	2	1	1	2	2	2	1	2	2	1	2
7. Loss to follow up less than 5%: all patients should be included in the follow up. Otherwise, the proportion lost to follow up should not exceed the proportion experiencing the major endpoint	2	2	2	2	2	2	2	1	2	2	2	2	2	2	2	2
8. Prospective calculation of the study size: information of the size of detectable difference of interest with a calculation of 95% confidence interval, according to the expected incidence of the outcome event, and information about the level for statistical significance and estimates of power when comparing the outcomes	0	0	1	0	0	0	0	2	0	0	0	0	0	0	0	0
**Additional criteria in the case of comparative study**																
9. An adequate control group: having a gold standard diagnostic test or therapeutic intervention recognized as the optimal intervention according to the available published data							0									0
10. Contemporary groups: control and studied group should be managed during the same time period (no historical comparison)							2									2
11. Baseline equivalence of groups: the groups should be similar regarding the criteria other than the studied endpoints. Absence of confounding factors that could bias the interpretation of the results							2									2
12. Adequate statistical analyses: whether the statistics were in accordance with the type of study with calculation of confidence intervals or relative risk							2									2
Total	8	9	13	9	8	7	15	11	9	8	7	6	10	8	7	14

### Rates of infectious eradication and complications

**Fig. 2:**
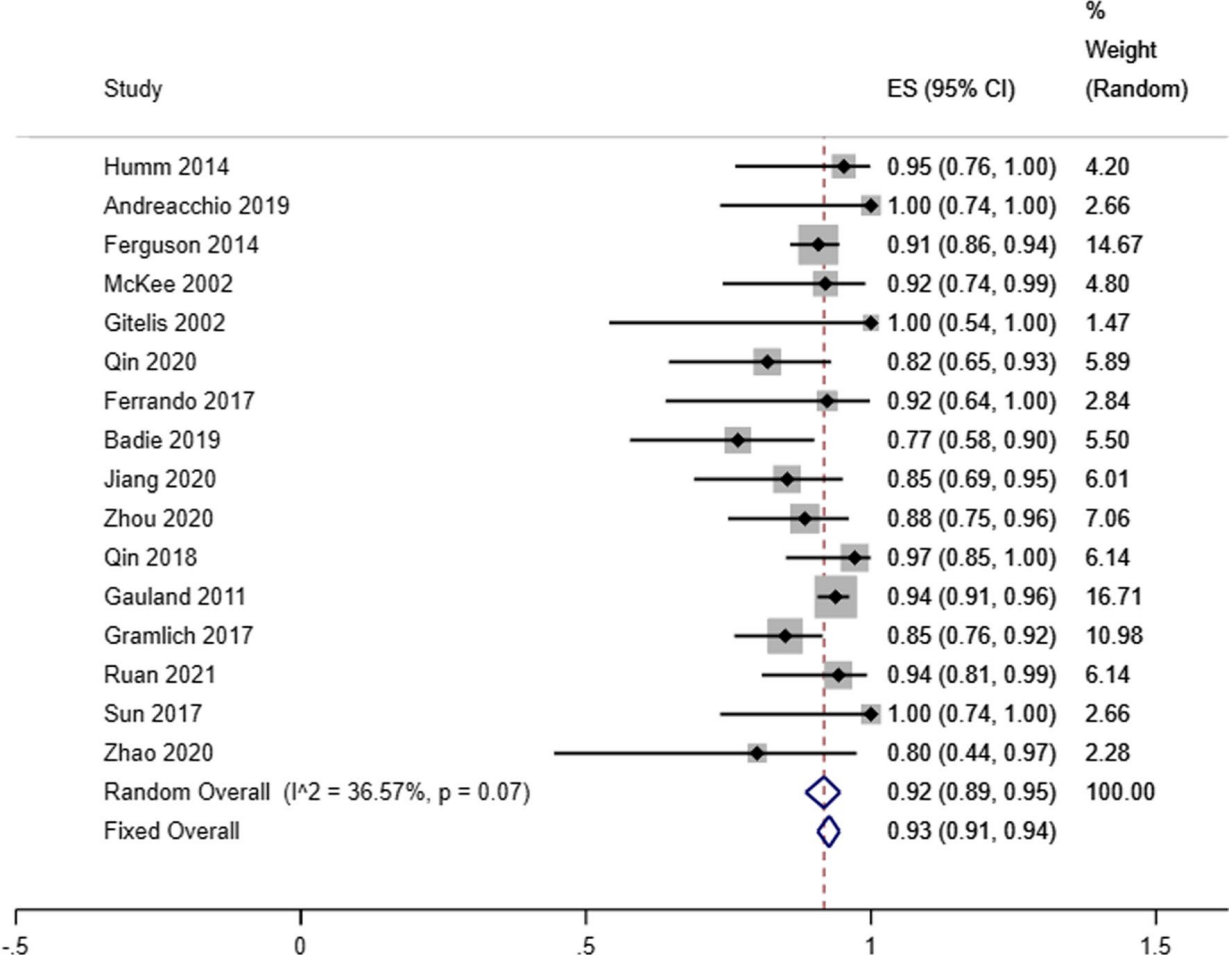
The overall eradication rate in COM patients with antibiotic-loaded calcium sulfate

**Fig. 3:**
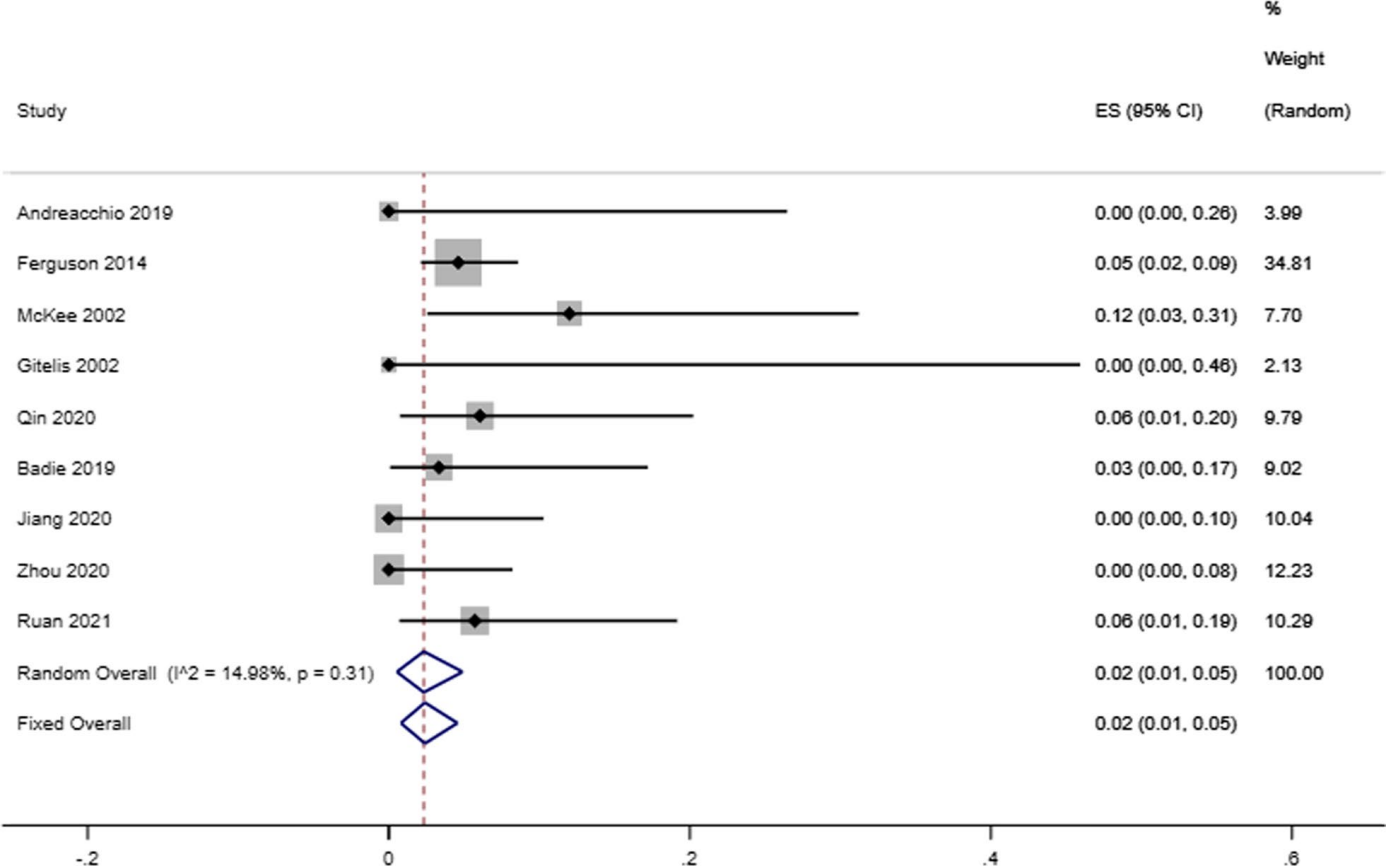
The overall refracture rate in COM patients with antibiotic-loaded calcium sulfate

**Fig. 4:**
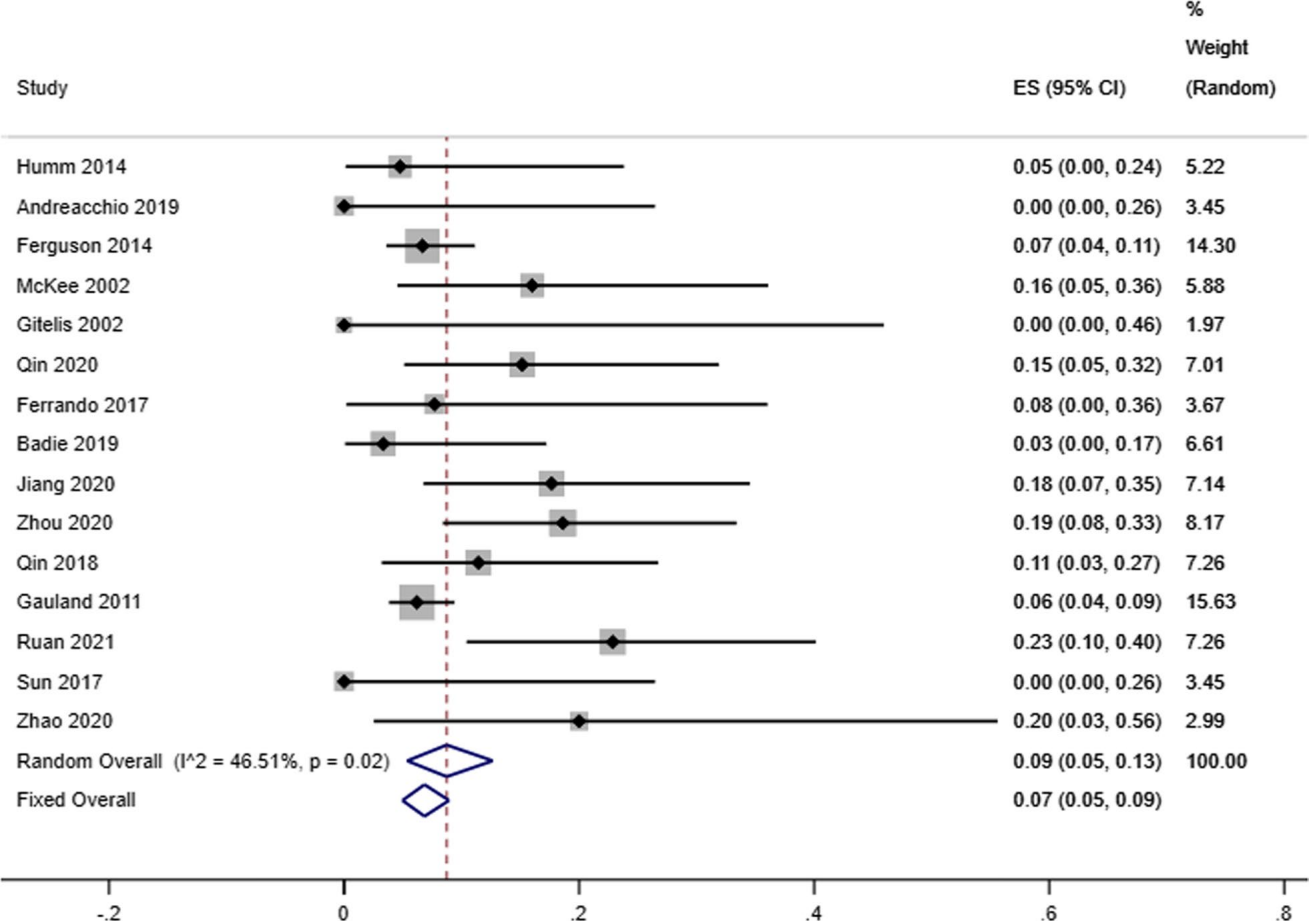
The overall reoperation rate in COM patients with antibiotic-loaded calcium sulfate

**Fig. 5:**
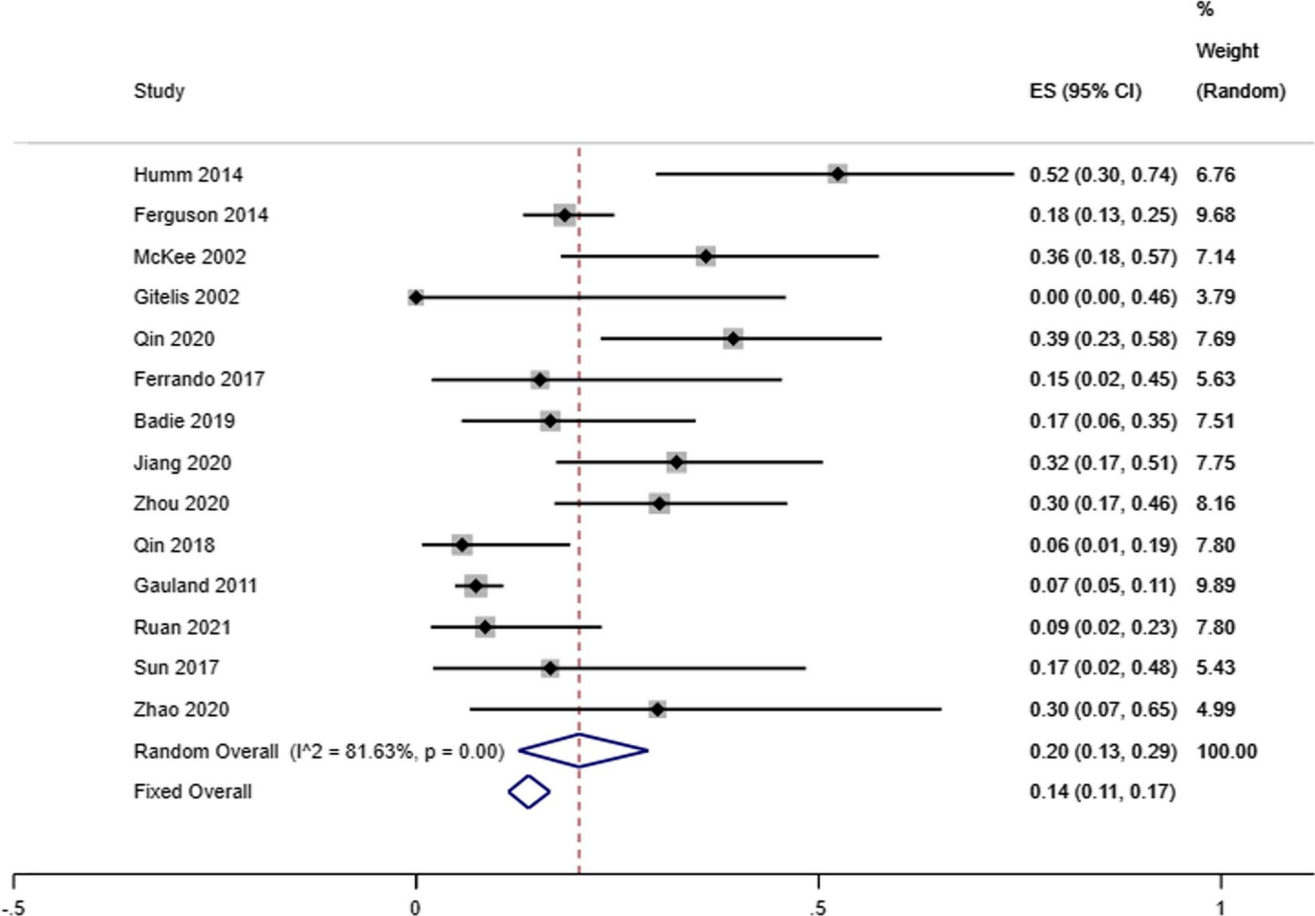
The overall rate of delayed healing in COM patients with antibiotic-loaded calcium sulfate

**Fig. 6:**
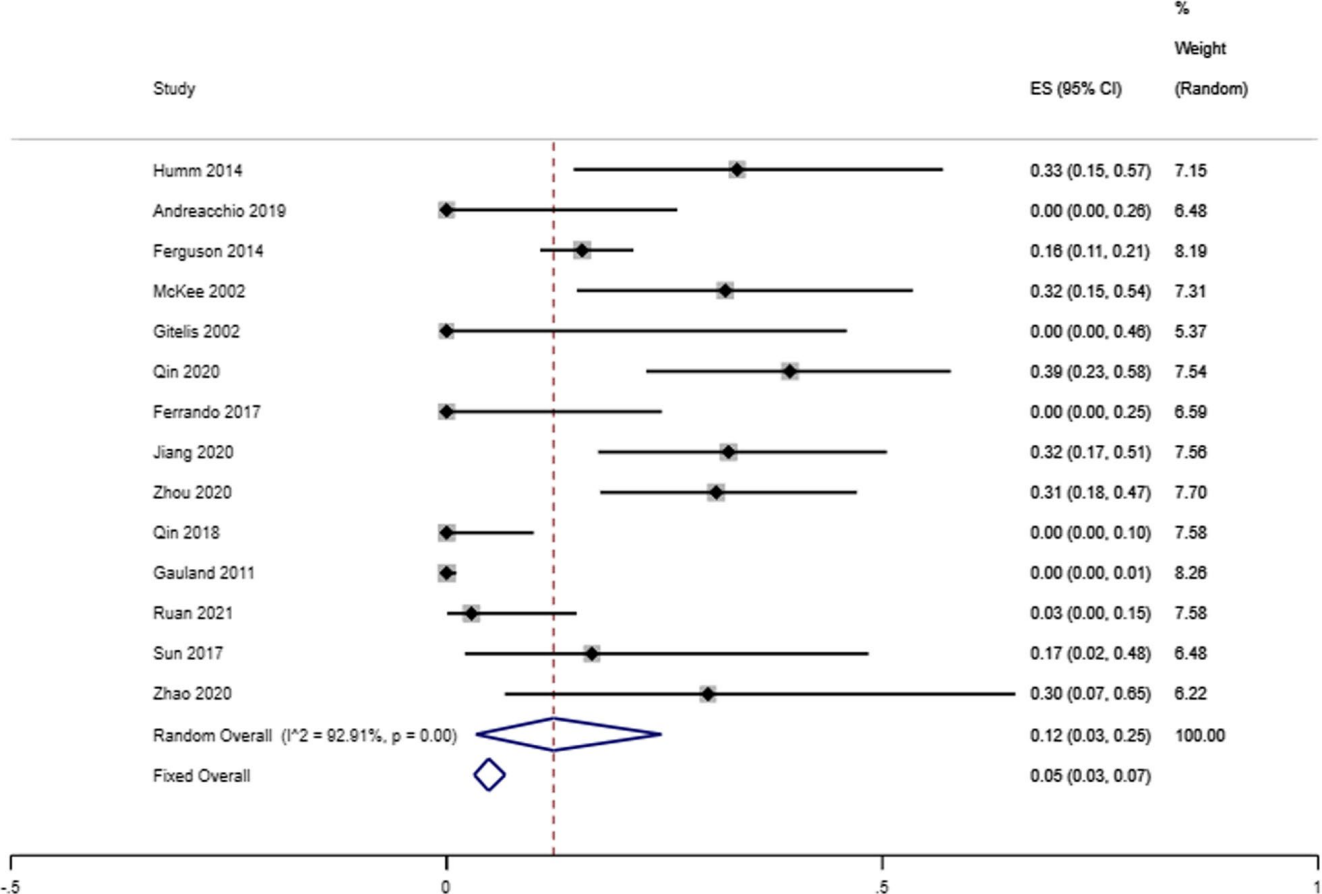
The overall rate of aseptic wound leakage in COM patients with antibiotic-loaded calcium sulfate

Our analysis showed that the infectious eradication rate among 717 patients (720 locations) receiving antibiotic-loaded calcium sulfate implantation was 92% reported by 16 studies (95% CI 0.89–0.95; *P* = 0.07), a random-effects model was used due to the low level of heterogeneity (*I*^2^ = 36.57%) (Fig. 2). A fixed effects model was used due to no heterogeneity (*I*^2^ = 23.96%), and the refracture rate was 2% in 9 studies (95% CI 0.00–0.04; *P* = 0.23) (Fig. 3). A random-effects model was used due to the low level of heterogeneity (*I*^2^ = 46.51%), and the reoperation rate was 9% reported by 15 studies that enrolled 827 cases4 (95% CI 0.05–0.13; *P* = 0.02) (Fig. 4). A random-effects model was used due to the high level of heterogeneity (*I*^2^ = 81.63%), and the delayed healing rate was 20% reported by 14 studies that enrolled 815 cases (95% CI 0.13–0.29; *P* = 0.001) (Fig. 5). A random-effects model was used due to the high level of heterogeneity (*I*^2^ = 92.91%), and the incidence of aseptic mouth leakage was 12% reported by 14 studies that enrolled 794 cases (95% CI 0.03–0.25; *P* = 0.001) (Fig. 6).

Among the 16 articles we included, we excluded one study that did not specify the location of COM [25]. The tibia was the most commonly affected part of COM and was detected in 46.73% of patients with COM (279/597). The second and third most common locations of COM were femur (139/597, 23.28%) and calcaneum (74/597, 12.40%), respectively. The remaining locations of COM included humerus (21/597, 3.52%), radius (12/597, 2.01%), pelvis (7/597,1.17%), fibula (7/597, 1.17%), ankle (6/597, 1.01%), hip joint (5/597, 0.84%), ulna (5/597, 0.84%), knee (4/597, 0.67%), talus (3/597, 0.50%), clavicle, forefoot, and fourth metatarsal each account for one case (1/597, 0.17%). A several COM patients had two involved locations [33, 37].

### Effects and complications of subgroup

In order to further explore the difference between the two kinds of antibiotics in the treatment of COM, the eradication rate and complications were summarized. However, the results showed that the eradication rates of tobramycin-loaded CS group and vancomycin combined with gentamicin-loaded CS group were 92% (95% CI 0.88–0.95; *P* = 0.9) and 90% (95% CI 0.86–0.94; *P* = 0.03), respectively, and there was no significant difference (*P* = 0.96). In terms of the incidence of complications, the reoperation rates of tobramycin-loaded CS and vancomycin combined with gentamicin-loaded CS were 7% (95% CI 0.04–0.10; *P* = 0.3) and 11% (95% CI 0.86–0.94; *P* = 0.03), the aseptic wound leakage rates were 24% (95% CI 0.11–0.38; *P* = 0.06) and 26% (95% CI 0.05–0.46; *P* = 0.001), the bone fracture rates were 5% (95% CI 0.02–0.08; *P* = 0.5) and 5% (95% CI 0.01–0.09%; *P* = 0.9), the incidence of delayed healing rates were 34% (95% CI 0.14–0.28; *P* = 0.001) and 17% (95% CI 0.13–0.55; *P* = 0.001), respectively. The results showed no significant difference (all *P* > 0.05) (Table 3).

**Table 3:** Summary of complications and efficacy outcomes in the included studies

	Tobramycin (95% CI)	Vancomycin and gentamicin (95% CI)	Sig (significant difference)
Eradication rate	0.92 (0.88, 0.95)	0.90 (0.86, 0.94)	0.373
Reoperation rate	0.07 (0.04, 0.10)	0.11 (0.06, 0.15)	0.497
Refracture rate	0.05 (0.02, 0.08)	0.05 (0.01, 0.09)	0.800
Delayed healing rate	0.34 (0.13, 0.55)	0.17 (0.10, 0.25)	0.327
Rate of aseptic wound leakage	0.24 (0.11, 0.38)	0.26 (0.05, 0.46)	0.857

### Publication bias

**Fig. 7:**
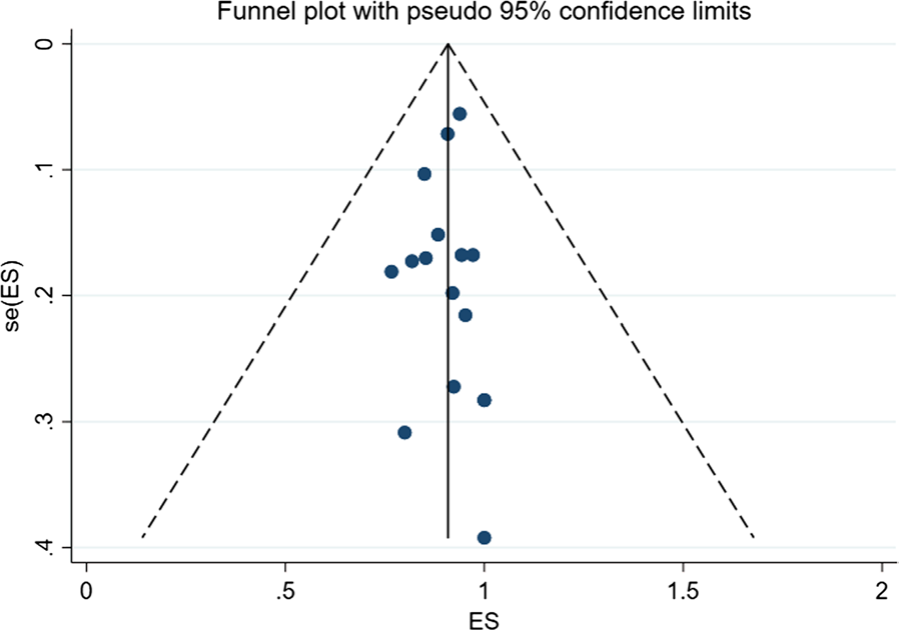
Funnel plot for assessing publication bias

A funnel chart would reflect whether there was publication bias in this study, and the symmetry of the funnel chart meant that there was no publication bias (Fig. 7). Furthermore, the symmetry test of the above graph showed that *P* = 0.882 > 0.05, which meant that the funnel graph was symmetrical. Therefore, it can be judged that there was no publication bias in current study.

### Sensitivity analysis

**Fig. 8:**
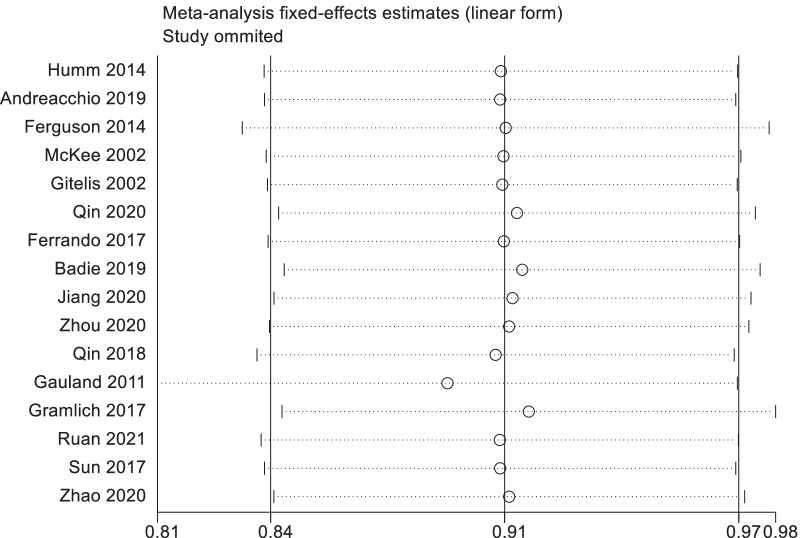
The results of sensitivity analysis

We eliminated each included study one by one, and summarized and analyzed the remaining studies to assess whether the individual study had an impact on the results of the meta-analysis. None of the studies had an excessive impact on the results of the meta-analysis, indicating that the results of the remaining studies were stable and reliable (Fig. 8).

## Discussion

CS was discovered in 1970 until it was used clinically in the past two decades, and achieved good results in Europe [15]. However, no research reported the specific efficacy of CS antibiotic delivery system in the treatment of patients with COM. In the current study, 16 studies published from 2000 to 2021 were included, with a total of 717 patients (720 locations) with COM. To the best of our knowledge, this was the first study to investigate the eradication rate in patients with COM, and we concluded that the overall rate of eradication in COM patients was 92%. The eradication rate was also similar to those reported for treatment with antibiotic-loaded PMMA beads in the literature, which showed 60% and 100% in between [42, 43].

The successful eradication of COM remains a challenge for clinicians. Except for a study on chronic jaw osteomyelitis that did not specify the use of antibiotics [1], the treatment regimen in all 15 studies involving the antibiotics-loaded CS included implantation after radical debridement followed by adjuvant systemic administration of antibiotics. Statistically, the tibia is the most common site for COM to occur [44], This is similar to our summary study. Of the 597 patients with COM included, nearly half were tibial osteomyelitis, because of its poor blood supply (especially inferior third of tibia), higher risk of injuries, and of course, the inappropriate surgical managements. Unfortunately, even standard treatment protocols have been strictly implemented, the recurrent rate of chronic tibial osteomyelitis remains as high as 20–30% [45]. Although we included 279 patients with tibial osteomyelitis, the recurrence of infection in each study was unable to determine whether it was on tibial or not, therefore made it difficult to calculate the recurrence rate of tibial osteomyelitis.

While infection elimination had shown encouraging results, associated local complications were also of concern. Delayed wound healing was the most frequent complication in our study, with a relatively high incidence of 20%. Bibbo et al. [46] reported that the delayed healing rate of calcium sulfate loaded with vancomycin in the treatment of calcaneal fractures was 15%, which was similar to our summary results. Kallala et al. [47] reported that aseptic wound leakage is a common complication of degradation of CS after implantation, which is related to the composition of CS itself, with an incidence of 4.2%, this incidence was variant with each individual, mainly depending on the size of implanted CS and the abundance of soft tissues coverage. Romano et al. [48] found that the leakage rate of aseptic wound was as high as 27%, while our total leakage rate of aseptic wound was 12%. This may be explained that some of the studies we included used different treatment methods after implantation of CS according to different conditions of patients, such as muscle flap coverage [39] or external fixation [29]. On the other hand, it may also be attributed to the fact that synthetic CS contains no impurities compared with mined and refined CS. At present, there is no reliable large-sample comparative study to confirm the side effects of calcium sulfate-induced leakage of sterile wounds. In addition to the wound leakage, local CS implantation may also bring other complications, such as heterotopic ossification [47] and even hypercalcemia [49]. Therefore we also pooled the refracture rate and the reoperation rate, which are 2% and 9%, respectively.

Antibiotic-loaded CS delivery system had been put into clinical application in the past 20 years, loaded with several of the most common antibiotics which made into a variety of products, such as vancomycin, gentamicin and tobramycin. However, the efficacy of calcium sulfate-loaded antibiotics in the treatment of COM is not clear. In an in vitro experiment, vancomycin and tobramycin impregnated materials had similar germicidal properties and elution efficiency [15]. A systematic review shows that there may be no significant difference between CS and other products except for degradation time [50]. The same was true for our subgroup results, we found that there was no significant difference in eradication rate between tobramycin loaded and vancomycin loaded with gentamicin calcium sulfate. However, there was no significant difference in the incidence of complications, including delayed healing rate, aseptic wound leakage rate, bone fracture rate and reoperation rate. That might be explained by different risk factors and patient individual variations.

Our study has some limitations. First, the sample size is not big enough and the summary of patient population characteristics is incomplete, which may hinder the interpretation of research results. Therefore, the results should be interpreted cautiously, and more patients should be enrolled in future studies to draw more precise conclusions. Second, we did not analyze the risk factors of infection relapse and complications because of most included studies’ retrospective design. To better identify potential risk factors, a good comparison of these antibiotics-loaded materials requires large-scale prospective clinical trials, especially multi-center joint studies; crucially, the potential risk of aseptic wound leakage after local CS implantation should be fully considered when patients receive this treatment. Future studies may focus on the risk factors of local complication and infection relapse following local CS implantation, as well as the efficacy of other substitute materials. Third, we all know that the release time and concentration of such antibiotic sustained-release systems are critical to the treatment of COM, but the studies we included lack a detailed description of the pharmacokinetics, which is not conducive to further compare the efficacy of several types of antibiotics. We should pay more attention to the clinical research on the species and drug resistance of COM bacteria in the future [51]. Fourth, the studies included different sites of infection (femur, tibia, humerus, radius, ulna, pelvis, and even calcaneus) and four types of Cierny-Mader staging. Therefore, our research inevitably lacks an in-depth discussion of a single type and location of COM. More detailed data is useful for evaluation of the efficacy of antibiotic CS on different parts of COM in future.

## Conclusion

In conclusion, this meta-analysis revealed that the eradication rate of implantation of antibiotic-loaded CS in the treatment of COM was as high as 92%, and the incidence of complications including delayed healing rate, aseptic wound leakage rate, refracture rate and reoperation rate were relatively low. Although there is no significant difference in efficacy and complications between the two antibiotic-loaded CS regimens in the treatment of COM. The clinical efficacy of antibiotic-loaded CS in the treatment of COM needs to be confirmed by further study.

## Data Availability

As a meta-analysis, all raw data of this study are extracted from ten included studies. The datasets supporting the conclusions of this article are available in the 16 included studies.

## Abbreviations

COM: Chronic osteomyelitis
CS: Calcium sulfate
PMMA: Polymethyl methacrylate
PICOS: Population, intervention, comparison, outcomes, and study
MINORS: Methodological items for non-randomized studies
CI: Confidence interval
